# Adaptive Exposure Optimization for Underwater Optical Camera Communication via Multimodal Feature Learning and Real-to-Sim Channel Emulation

**DOI:** 10.3390/s25206436

**Published:** 2025-10-17

**Authors:** Jiongnan Lou, Xun Zhang, Haifei Shen, Yiqian Qian, Zhan Wang, Hongda Chen, Zefeng Wang, Lianxin Hu

**Affiliations:** 1 School of Information Engineering, Huzhou University, Huzhou 313000, China; 2022388324@stu.zjhu.edu.cn (J.L.); 2023388239@stu.zjhu.edu.cn (H.S.); 2023388107@stu.zjhu.edu.cn (Y.Q.); 2023388135@stu.zjhu.edu.cn (Z.W.); 03261@zjhu.edu.cn (H.C.); zefeng.wang@zjhu.edu.cn (Z.W.); 2 LISIT-ECoS, Institut Supérieur d’Électronique de Paris, 75006 Paris, France

**Keywords:** underwater optical camera communication, adaptive exposure, optical channel emulation, multimodal feature learning, CMOS imaging

## Abstract

Underwater Optical Camera Communication (UOCC) has emerged as a promising paradigm for short-range, high-bandwidth, and secure data exchange in autonomous underwater vehicles (AUVs). UOCC performance strongly depends on exposure time and International Standards Organization (ISO) sensitivity—two parameters that govern photon capture, contrast, and bit detection fidelity. However, optical propagation in aquatic environments is highly susceptible to turbidity, scattering, and illumination variability, which severely degrade image clarity and signal-to-noise ratio (SNR). Conventional systems with fixed imaging settings cannot adapt to time-varying conditions, limiting communication reliability. While validating the feasibility of deep learning for exposure prediction, this baseline lacked environmental awareness and generalization to dynamic scenarios. To overcome these limitations, we introduce a Real-to-Sim-to-Deployment framework that couples a physically calibrated emulation platform with a Hybrid CNN-MLP Model (HCMM). By fusing optical images, environmental states, and camera configurations, the HCMM achieves substantially improved parameter prediction accuracy, reducing RMSE to 0.23–0.33. When deployed on embedded hardware, it enables real-time adaptive reconfiguration and delivers up to 8.5 dB SNR gain, surpassing both static-parameter systems and the prior CNN baseline. These results demonstrate that environment-aware multimodal learning, supported by reproducible optical channel emulation, provides a scalable and robust solution for practical UOCC deployment in positioning, inspection, and laser-based underwater communication.

## 1. Introduction

As ocean exploration and marine resource utilization continue to advance, Autonomous Underwater Vehicles (AUVs) have become indispensable, particularly when deployed in swarms that enhance scalability, resilience, and cost-effectiveness [1]. These multi-agent systems are widely applied in subsea infrastructure inspection, environmental monitoring, and cooperative exploration of ecologically sensitive regions [2]. Effective coordination of such tasks requires a communication framework with high data throughput and low latency. While acoustic communication remains the dominant underwater standard due to its long range, it inherently suffers from limited bandwidth, large latency, and strong multipath interference—constraints that severely affect densely networked swarms [3,4]. In contrast, Underwater Optical Camera Communication (UOCC) has emerged as a promising short-range alternative that exploits the physical advantages of light propagation in water to provide high-bandwidth and low-latency links [5]. UOCC employs modulated light signals transmitted by LEDs and detected by Complementary Metal-Oxide-Semiconductor (CMOS) cameras, which are already integral to most AUV platforms for visual navigation. This dual-use capability minimizes hardware overhead and lowers deployment cost [6,7,8,9]. From an optical perspective, CMOS-based UOCC systems benefit from their large photosensitive area and wide field-of-view (FOV), enabling robust photon capture without stringent alignment. This provides critical flexibility under dynamic and unpredictable swarm configurations in open water. These unique optical advantages establish UOCC as a scalable and practical paradigm for the next generation of underwater robotic networks [10,11] and motivate the present study from the standpoint of optical communication system design.

Despite these advantages, the performance of UOCC systems remains highly sensitive to underwater channel disturbances, including turbidity, flow-induced scattering, optical absorption, and fluctuations in ambient illumination [12]. Such factors reduce photon transmission efficiency and distort the spatial contrast of modulated light patterns captured by CMOS sensors, resulting in a degraded signal-to-noise ratio (SNR), reduced data throughput, and, in severe cases, complete link outage [5,13]. A critical determinant of optical signal fidelity in camera-based receivers is the configuration of imaging parameters—in particular, the exposure time and International Standards Organization (ISO) sensitivity. These parameters directly govern photon capture, brightness, and contrast of the received stripe-based modulation patterns. However, their optimal values vary nonlinearly with environmental conditions such as scattering strength, absorption coefficient, and background illumination. This nonlinear and dynamic dependence makes manual adjustment impractical for real-world deployments, especially in mobile and autonomous platforms [14,15,16,17]. Recent advances in data-driven optical parameter control provide a promising direction. By learning the complex mapping between environmental states and imaging performance, machine learning models can dynamically adapt exposure and ISO settings, thereby maintaining robust photon acquisition and reliable decoding across diverse and time-varying underwater conditions.

Recent advances at the intersection of computational optics and machine learning have introduced deep learning-based approaches for intelligent image enhancement and signal optimization. Convolutional neural networks (CNNs), in particular, have demonstrated strong capabilities in processing spatially structured optical data, making them effective for mitigating scattering-induced blur and enhancing the visibility of modulation patterns in vision-based systems [18]. In parallel, deep reinforcement learning (DRL) has been explored as a framework that integrates perception and control, enabling adaptive responses to dynamic optical channels through continuous interaction [19]. These learning-based strategies have been increasingly applied to CMOS sensors, where they improve photon capture efficiency, contrast restoration, and optical signal recovery under diverse imaging conditions [20,21,22,23]. Within the specific context of underwater optical camera communication (UOCC), such methods are particularly valuable for preserving image clarity and enabling reliable decoding of modulated light signals in turbid or low-light environments. Prior studies have shown that deep learning can suppress sensor noise, compensate for scattering artifacts, and enhance the robustness of optical links under channel disturbances [24,25].

Building on these advances, our earlier work [26] proposed a CNN-based joint regression framework that fuses image features with parameter metadata to predict optimal exposure time and ISO in UOCC. This baseline model, employing a ResNet50 backbone and a parameter-aware regression head, demonstrated that data-driven parameter tuning can significantly improve decoding robustness, achieving a root mean square error (RMSE) of 0.96 and enhancing the synthetic SNR from 4.16 dB to 6.91 dB. While these results validated the feasibility of deep learning for adaptive imaging, the framework exposed three key limitations: (i) lack of explicit modeling of environmental disturbances, leading to unstable performance under turbidity or illumination fluctuations; (ii) reliance on static datasets without physical calibration, which restricted generalization beyond laboratory conditions; and (iii) absence of real-time deployment validation, limiting its applicability for embedded AUV platforms. These deficiencies highlight the gap between proof-of-concept CNN models and the requirements of practical UOCC deployments and directly motivate the present study to move beyond image–parameter fusion toward environment-aware multimodal learning under physically faithful emulation.

A major challenge in advancing UOCC through artificial intelligence lies in the limited availability of labeled datasets for model training. In optical communication research, this issue has often been addressed via synthetic data generation. For example, refs. [27,28] employed simulated channel parameters to train CNN-based channel estimators, while refs. [29,30] developed custom testbeds for evaluating learning-based demodulation strategies. However, unlike free-space optical links, underwater optical channels exhibit far greater complexity due to the combined effects of multiple scattering, wavelength-dependent absorption, turbulence, and nonstationary illumination. These interactions are difficult to capture through purely mathematical models and require empirical validation to ensure physical relevance. To this end, researchers have constructed controlled laboratory platforms that permit systematic adjustment of environmental parameters such as turbidity and flow conditions [31]. Mechanical actuators—including propellers, water jets, and pumps—are often employed to mimic dynamic turbulence [32,33], while particulate additives such as Maalox, kaolin, or suspended sediments are used to emulate scattering-induced attenuation [34,35]. Although these testbeds provide valuable insights, achieving consistent and repeatable underwater channel states remains difficult. By contrast, field trials in natural aquatic environments offer higher realism but introduce substantial barriers, including high logistical costs, specialized instrumentation, and the unpredictability of environmental variability [36,37,38]. Consequently, the development of a practical, reproducible, and optically faithful emulation framework that can capture the inherent complexity of underwater light propagation under controlled conditions remains an unresolved challenge. Addressing this gap is crucial for bridging the divide between computational learning techniques and physically consistent optical channel modeling in UOCC.

In practical deployments, UOCC must also contend with dynamic and unpredictable conditions that preclude static camera settings. Adjusting exposure time and ISO sensitivity provides an efficient means of enhancing received SNR [12], especially for embedded systems on resource-limited platforms. While rule-based and data-driven approaches have been proposed [39], most were not designed specifically for underwater optical communication. Evidence from related OCC domains illustrates the benefits of adaptive control: indoor non-line-of-sight OCC systems achieve improved demodulation through dynamic parameter tuning [40], while UAV-based OCC employs recurrent neural models to mitigate motion-induced instability [5]. These findings align with UOCC environments, where turbidity fluctuations, flow-induced scattering, and illumination variability produce nonstationary optical disturbances. Yet open challenges remain: (1) the coupled impact of turbidity, flow, and lighting is analytically intractable; (2) real-world data collection is costly and non-repeatable, limiting labeled datasets; (3) models trained on simulated or ideal conditions often fail to generalize due to domain mismatch.

To address these challenges, we propose a Real-to-Sim-to-Deployment framework for adaptive UOCC. In this paradigm, real-world measurements calibrate a controlled laboratory emulator (Real-to-Sim), which supports training of a multimodal deep learning model for embedded deployment (Sim-to-Deployment). Our testbed employs 470 nm blue LEDs and CMOS cameras with adjustable Exposure Values (EV), under turbidity levels tunable from 100 to 400 NTU, enabling reproducible emulation of scattering and absorption. Using this dataset, we design a hybrid CNN-MLP model (HCMM) that integrates visual features, environmental sensor inputs, and camera configuration states to predict optimal exposure and ISO values. Compared with our prior CNN-based baseline, the proposed multimodal framework explicitly incorporates environment-aware features and achieves substantially higher robustness, delivering up to 8.5 dB SNR gain under dynamic conditions. Deployed in field trials, the system provides real-time inference and adaptive reconfiguration, demonstrating scalability for AUV-based UOCC.

The main contributions of this work are as follows:

1. Real-to-Sim-to-Deployment framework: a hierarchical transfer approach that bridges empirical underwater data and simulation-based training, enabling adaptive UOCC deployment on AUVs.
2. Sensor-integrated emulation platform: a controllable and reproducible testbed that standardizes benchmarking of UOCC under variable turbidity, flow, and illumination.
3. Hybrid CNN–MLP model (HCMM): extending our prior CNN-based baseline, we develop a multimodal learning architecture that fuses image, sensor, and configuration features to predict optimal parameters, significantly improving underwater optical communication robustness.

## 2. Proposed Method and Principle

**Figure 1:**
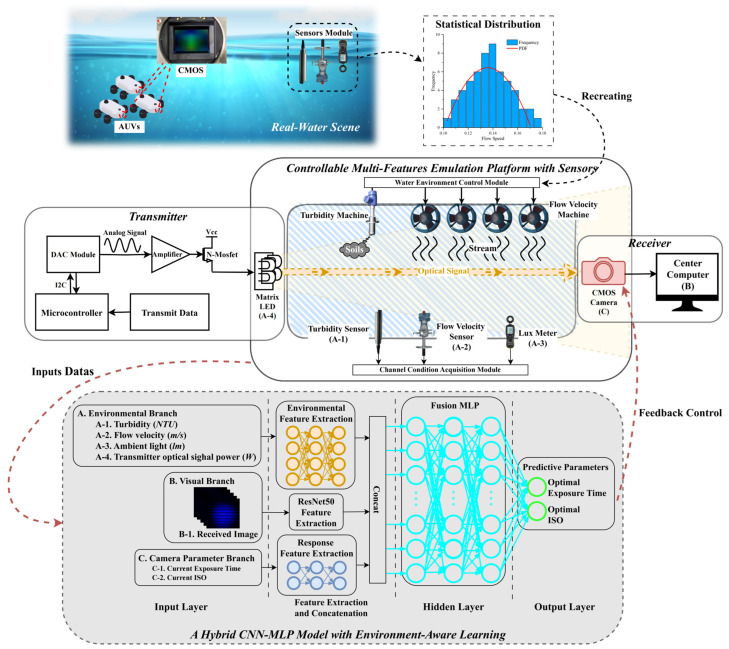
The proposed method and system framework.

To bridge the performance gap between controlled laboratory experiments and the complex variability of real underwater environments, we propose a Real-to-Sim-to-Deployment framework for adaptive imaging parameter control in underwater optical camera communication (UOCC). Unlike conventional sim-to-real strategies, where models are trained in synthetic environments and then fine-tuned with real data, our approach adopts an inverse methodology. Specifically, real-world underwater measurements are first used to calibrate a controlled laboratory environment, ensuring that critical channel characteristics such as scattering, turbidity, and illumination variability are faithfully reproduced. This calibration enhances the realism of simulated datasets and improves the generalizability of trained models. Consequently, deep learning models developed in this framework can be deployed directly in AUV platforms with minimal fine-tuning. The overall architecture of the proposed method is illustrated in Figure 1.

Figure 1 illustrates the overall framework comprising three key modules. The Controllable Multi-Features Emulation Platform with Sensors recreates underwater optical conditions by adjusting turbidity, flow velocity, and illumination while collecting synchronized sensor data. The transmitter generates modulated optical signals using a microcontroller, DAC, amplifier, and N-MOSFET to drive a matrix LED. The Hybrid CNN-MLP Model with Environment-Aware Learning fuses environmental, visual, and camera-state features to predict optimal exposure and ISO parameters for adaptive feedback control in UOCC.

The process begins with real-world data acquisition across diverse underwater conditions, covering variations in turbidity, flow velocity, and ambient illumination. Instead of serving directly as training data, these measurements are statistically analyzed to derive probability distribution functions (PDFs) that capture temporal and spatial fluctuations of environmental parameters. These PDFs act as statistical priors, guiding the construction of a sensor-integrated emulation platform. This platform reproduces representative underwater disturbances such as scattering and absorption under controlled, repeatable conditions. By generating large-scale labeled datasets that are otherwise impractical to collect in field trials, the platform enables systematic training and validation of learning-based models under realistic optical disturbances.

The hardware architecture consists of two subsystems: a transmitter and a configurable emulation platform. The transmitter incorporates a digital-to-analog converter (DAC), bias control, and interchangeable LEDs to support variable amplitude and optical source conditions. The emulation platform integrates a turbidity control module, flow-velocity generators, and illumination regulators, all monitored by embedded sensors (turbidity, flow, and lux). These modules allow precise control and feedback, ensuring accurate recreation of diverse underwater optical channels. At the receiver side, the optical signals are captured by either a photodetector (PD) for sensitivity benchmarking or a CMOS camera for imaging-based experiments. The received images and sensor data form a multimodal dataset, directly linking environmental conditions with optimal camera parameter configurations.

To exploit this dataset for real-time parameter adaptation, we design a hybrid CNN-MLP model (HCMM) that integrates multimodal feature learning. The architecture consists of three dedicated encoding branches:

- Visual branch: a convolutional neural network (CNN) extracts high-level features from the received image, capturing distortions induced by scattering and illumination.
- Environmental branch: low-dimensional sensor data (turbidity, flow velocity, illumination, and transmitter power) are encoded to represent channel states.
- Camera parameter branch: current exposure and ISO values are provided as context to stabilize predictions.

Features from the three branches are fused through a multilayer perceptron (MLP), which outputs the predicted optimal exposure time and ISO settings. By explicitly combining optical channel modeling with data-driven feature fusion, the HCMM achieves robust cross-modal learning and provides adaptive imaging control tailored to the highly dynamic conditions of UOCC.

### 2.1. Visual Branch

To extract high-level semantic features from underwater optical images, we employ a visual processing branch based on a fixed ResNet50 architecture pretrained on ImageNet. ResNet50 is a deep convolutional neural network comprising convolutional layers, residual blocks, and a global average pooling layer. Its core innovation lies in residual learning, where each block learns a residual function *F(x)*, allowing the network to model the target function as

**Figure 2:**
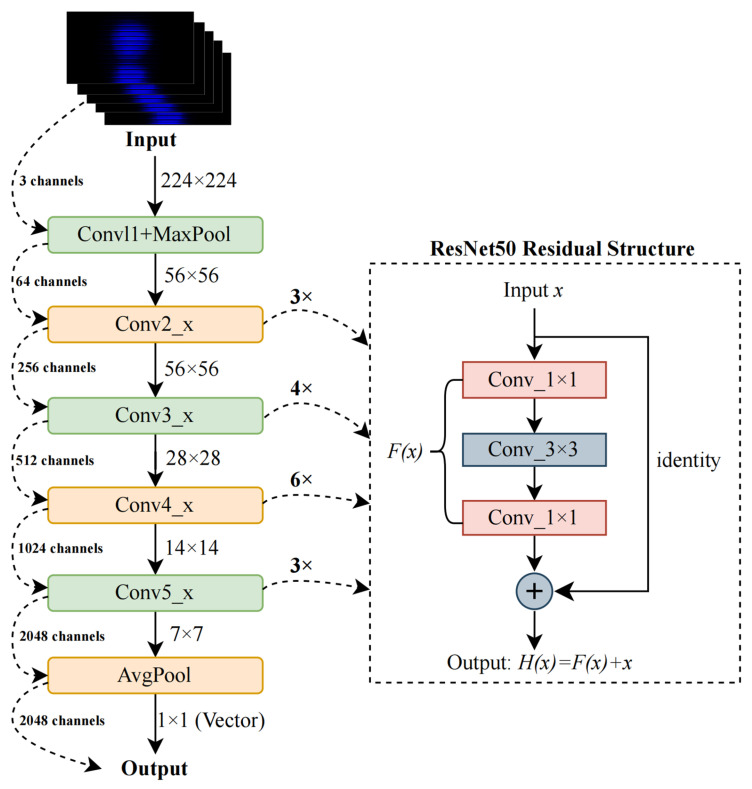
Dimensional transformation between layers.

(1) H(x)=F(x)+x

where *x* is the input to the residual block, *F*(*x*) is the learned residual function (typically composed of convolutional layers and nonlinear activations), and *H*(*x*) is the final output of the block. This structure mitigates vanishing gradients and facilitates stable training in deep networks. The residual design enables the network to preserve low-level details while capturing complex patterns, making it well-suited for modeling structured visual cues in underwater imagery. A typical residual block structure, including skip connections and convolutional operations, is illustrated in Figure 2. Input images, originally sized at 1920 × 1080, are resized to 224 × 224 × 3 and normalized using ImageNet statistics before being fed into the network. ResNet50 then processes the image through a hierarchical series of convolutional and residual layers, gradually increasing feature abstraction. The transformation of feature dimensions across layers is also depicted in Figure 2.

Figure 2 details the internal structure of the visual branch based on ResNet50. It shows how input images (1920 × 1080 pixels) are resized, normalized, and successively processed through convolutional and residual blocks. The ResNet50 backbone processes the input image through a series of convolutional and residual layers, progressively increasing semantic abstraction. The image first passes through a 7 × 7 convolution with a stride of 2, followed by batch normalization, ReLU activation, and a 3 × 3 max pooling layer, producing a 56 × 56 × 64 feature map. Four residual block groups follow. The first group (Conv2_x) contains three blocks with 1 × 1, 3 × 3, and 1 × 1 convolutions, maintaining the 56 × 56 resolution and expanding the depth to 256 channels. The second group (Conv3_x) consists of four blocks with downsampling, reducing the resolution to 28 × 28 and increasing the depth to 512. The third group (Conv4_x) includes six blocks, yielding a 14 × 14 × 1024 feature map. The final group (Conv5_x) has three blocks, producing a 7 × 7 × 2048 output. A global average pooling layer condenses this high-dimensional output into a 2048-dimensional vector, capturing the most salient visual features. To align with other modality embeddings and ensure stable training, this vector is projected through a linear layer to obtain a 512-dimensional latent representation, which serves as the visual feature input for the fusion stage, which can be expressed as

(2) $$v_{visual}=ReLU(W_{proj}\cdot z_{img}+b)\in R^{512}$$

where $z_{img}$ denotes the visual feature vector extracted from the ResNet50 encoder; $W_{proj}\;$ and b denote the learnable projection matrix and bias vector, respectively; and $v_{visual}\in R^{512}$ denotes the projected and activated visual embedding obtained after the ReLU operation. The 512-dimensional visual representation is concatenated with embeddings from the environment and camera parameter branches and fed into a joint fusion network. This design allows the model to integrate visual cues with physical context, enabling exposure and ISO predictions that are both perceptually relevant and environmentally adaptive.

### 2.2. Environment Branch

To model the impact of environmental disturbances during image acquisition, we introduce a compact encoding module that processes physical water conditions. The module receives a normalized four-dimensional input vector comprising turbidity (NTU), flow speed (m/s), LED transmission power (W), and ambient illumination (lm), each representing a key source of interference in UOCC systems.

To ensure numerical stability and effective learning across these heterogeneous inputs, all parameters are normalized using min–max scaling. Each value is rescaled by subtracting the minimum of its range and dividing by its span, ensuring balanced contribution across dimensions. The resulting normalized scalars form a four-dimensional environmental feature vector, defined as

(3) $$X_{env}=[Turbidity,\;Flowspeed,\;lightpower,Ambientlight]\in R^{4}$$

where $X_{env}$ denotes the environmental parameter vector consisting of four physical factors: turbidity, flow speed, light power, and ambient light intensity, each normalized to [0,1]; thus, $X_{env}$ represents the environmental state input to the model. The resulting vector provides a numerically balanced representation of the environmental context for each image sample. This four-dimensional normalized input is processed by a compact neural encoder designed to capture nonlinear relationships among the variables. The encoder consists of a fully connected layer that projects the input into a 64-dimensional latent space, followed by a ReLU activation and Layer Normalization to promote stable and efficient training. This transformation can be expressed as

(4) $$v_{e}=LayerNorm(ReLU(W_{e}\cdot X_{env}+b_{e}))\in R^{64}$$

where $X_{env}$ denotes the 4D environmental input vector; $W_{e}$ and $b_{e}$ denote the learnable projection matrix and bias; ReLU(·) denotes the element-wise rectified linear unit; LayerNorm(·) denotes layer normalization applied to the projected features; and $v_{e}$ denotes the normalized environmental embedding. The resulting 64-dimensional embedding captures the latent influence of environmental conditions on the imaging process.

### 2.3. Camera Parameter Branch

In addition to modeling scene content and environmental conditions, the framework includes a camera parameter encoding branch to represent the intrinsic configuration of the imaging system. Specifically, it accounts for the current exposure time and ISO level—two key factors influencing sensor sensitivity and signal response under varying lighting. A normalized two-dimensional input vector [Curr_Exp, Curr_ISO] captures the camera state at the time of image acquisition. This vector is passed through a fully connected layer to project it into a 64-dimensional latent space, followed by a ReLU activation to model nonlinear effects such as sensor saturation and amplification. This transformation can be expressed as

(5) $$v_{c}=ReLU(W_{c}\cdot p_{cam}+b_{c})\in R^{64}$$

where $p_{cam}$ denotes the two-dimensional camera state vector composed of the current exposure level and ISO value; $W_{c}$ denotes the learnable weight matrix and bias term of the camera branch; and $v_{c}$ denotes the resulting camera feature embedding. The camera-state encoding is modeled as an independent branch, separate from environmental and visual inputs. This modular design reflects the physical separability of the parameters: exposure and ISO are internal system settings, while environmental factors are externally driven. Decoupling their representations prevents entanglement, allowing the model to learn their effects more accurately and improving prediction reliability across both low-SNR and overexposed conditions.

### 2.4. Fusion and Hidden Layers

To enable joint reasoning over visual content, environmental context, and device configuration, we implement a multimodal fusion mechanism that combines the latent features from the three independent branches. Specifically, the 512-dimensional visual feature vector, the 64-dimensional environmental embedding, and the 64-dimensional camera-state representation are concatenated to form a unified representation, which can be expressed as

(6) $$v_{fusion}=Concat(v_{visual},v_{e},\;v_{c}\;)\in R^{640}$$

where and Concat*(·)* denotes channel-wise concatenation along the feature dimension, yielding the fused representation $v_{fusion}$. The fused vector is passed through a three-layer fully connected network (Fusion MLP) designed to capture nonlinear interactions across modalities. The first hidden layer applies a linear transformation, followed by ReLU activation and dropout regularization, producing a 256-dimensional intermediate representation:

(7) $$z_{1}=ReLU(W_{1}\cdot v_{fusion}+b_{1}),\;z_{1}\in R^{256}$$

followed by ${\tilde{z}}_{1}=Dropout(z_{1},\;p)$ to prevent overfitting. The second and third hidden layers further map the feature space to 128 dimensions, each followed by ReLU activation:

(8) $$z_{2}=ReLU(W_{2}{\tilde{z}}_{1}+b_{2}),\;z_{2}\in R^{128}$$

(9) $$z_{3}=ReLU(W_{3}z_{2}+b_{3}),\;z_{3}\in R^{128}$$

(10) $$[{\hat{e}}_{opt},\;{\hat{i}}_{opt}]=W_{out}z_{3}+b_{out}$$

where $W_{1}$, $W_{2}$, $W_{3}$, $W_{out}$ and $b_{1}$, $b_{2}$, $b_{3}$, $b_{out}$ denote the learnable weight matrices and bias terms of the multilayer perceptron (MLP) head; $Dropout(z_{1},p)$ denotes random feature dropout with a probability *p* to prevent overfitting; $z_{1}$, $z_{2}$, $z_{3}$ denote the hidden-layer feature vectors of dimensions 256, 128, and 128, respectively; and [${\hat{e}}_{opt},\;{\hat{i}}_{opt}$] denote the predicted normalized optimal exposure and ISO outputs of the model. Finally, the output layer applies a linear transformation to produce a two-dimensional regression output, representing the predicted normalized exposure time (Opt_Exp) and ISO (Opt_ISO) for the given scene. These values correspond to the HCMM’s optimal camera parameter estimates under current environmental and visual conditions.

### 2.5. Algorithm

To clearly describe the data flow and computational pipeline of the proposed Hybrid CNN–MLP Model (HCMM), the complete training and deployment process is summarized in Algorithm 1. This algorithm outlines the sequential operations from data preprocessing and feature extraction to multimodal fusion, optimization, and real-time inference, ensuring reproducibility and easy implementation on embedded platforms.
**Algorithm 1:** Adaptive Exposure Optimization via HCMM**Input**: RGB image/(1920 × 1080), environment vector e = [Turbidity, Flow, Power, Lux], camera state c = [Curr_Exp, Curr_ISO]$\mathrm{Output}:\;\hat{y}$ = *[*
${\hat{e}}_{opt},\;{\hat{i}}_{opt}$
*]*
1: Resize I→224×224, normalize (lmageNet stats)2: Normalize *e*, *c* using ranges in Equations (3) and (5)$3:\;\mathrm{Visual}\;\mathrm{branch}:\;v_{visual}=ReLU(W_{proj}\cdot z_{img}+b)\in R^{512}$$4:\;\mathrm{Env}\;\mathrm{branch}:\;X_{env}=[Turbidity,\;Flowspeed,\;lightpower,Ambientlight]\in R^{4}$ $\;v_{e}=LayerNorm(ReLU(W_{e}\cdot X_{env}+b_{e}))\in R^{64}$$5:\;\mathrm{Cam}\;\mathrm{branch}:\;v_{c}=ReLU(W_{c}\cdot p_{cam}+b_{c})\in R^{64}$$6:\;\mathrm{Fusion}:\;v_{fusion}=Concat(v_{visual},v_{e},\;v_{c}\;)\in R^{640}$$7:\;\mathrm{MLP}\;\mathrm{head}:\;z_{1}=ReLU(W_{1}z_{0}+b_{1}),\;z_{1}\in R^{256}$$;{\tilde{z}}_{1}=Dropout\;(z_{1},\;p)$*;* $z_{2}=ReLU(W_{2}{\tilde{z}}_{1}+b_{2}),\;z_{2}\in R^{128}$$;z_{3}=ReLU(W_{3}z_{2}+b_{3}),\;z_{3}\in R^{128}$*;* $\hat{y}\;=\;W_{out}z_{3}+b_{out}\in R^{2}$$8:\;\mathrm{Train}\;\mathrm{with}\;\mathrm{MSE},\;\mathrm{Adam}(1\;\times \;{10^{-}}^{4}\to$1 × 10^−5^), early-stopping$9:\;\mathrm{Deploy}:\;\mathrm{clamp}\;\hat{y}$ to valid EV/lSO ranges; update camera driver

## 3. Experimental Setup

### 3.1. Overview of the Experimental Test Bench

**Figure 3:**
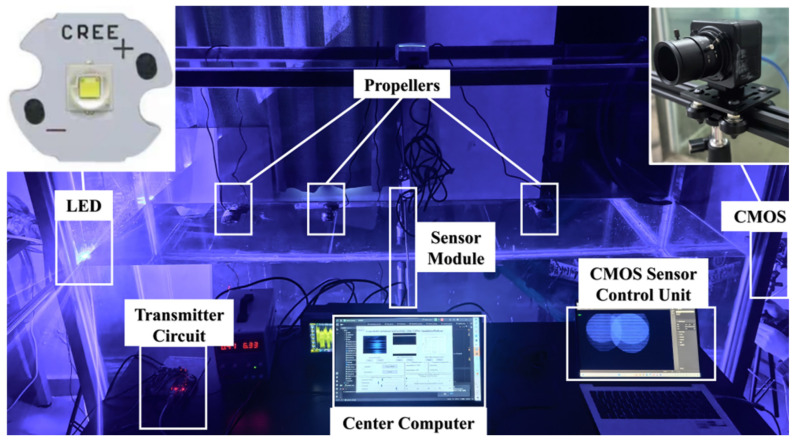
Schematic diagram of the laboratory testing platform.

The proposed method was experimentally validated in a controlled laboratory environment, as shown in Figure 3. The setup featured a transparent glass water tank with internal dimensions of 200 cm × 40 cm × 75 cm (excluding the 0.5 cm glass thickness). An optical transmitter and a CMOS camera-based receiver were mounted externally at the centers of the opposing short sides, forming a 2-m underwater optical communication link. This configuration allowed for repeatable signal transmission and image acquisition under simulated aquatic conditions.

To emulate real-world underwater disturbances, three submersible propellers (Model YVP-03B, 7 W, 4000 L/h, Shenling, Ningbo, China) were used to generate turbulence, and calibrated amounts of clay were added to vary turbidity. Real-time environmental monitoring was achieved using three industrial-grade sensors placed at the tank’s center:

1. Turbidity sensor (LS300A, Ouka, Nanjing, China): Based on optical scattering, with 0.001 NTU resolution, 0–1000 NTU range, and ±2% typical accuracy.
2. Flow velocity sensor (LS002351, Zhongyi Technology, Guangzhou, China): Uses a rotating propeller and encoder and supports 0.01–4 m/s measurements, with ±5% accuracy.
3. Ambient light sensor (XM7663M, STMicroelectronics, Geneva, Switzerland): Submersible, 380–730 nm spectral range, 0.01 LUX resolution, 0–600 LUX range, and ±5% accuracy.

Although saline water changes the refractive index and increases red-wavelength absorption, our 470 nm source lies near a low-absorption window, so we expect qualitatively similar trends; validation in brackish and seawater is deferred to Section 5.2. Overall, the platform provides fine, reproducible control of flow, turbidity, and illumination, enabling consistent data collection and rigorous assessment of model performance across diverse, yet well-specified, conditions.

Figure 3 illustrates the Real-to-Sim UOCC test bench: a CREE blue LED driven by a programmable transmitter circuit, three submersible propellers for tunable flow, a mid-tank sensor module for turbidity/flow/illumination, a CMOS camera with control unit, and a center computer generating PRBS and logging data. The 2-m-long water path enables repeatable scattering/turbulence for physically consistent dataset collection.

**Figure 4:**
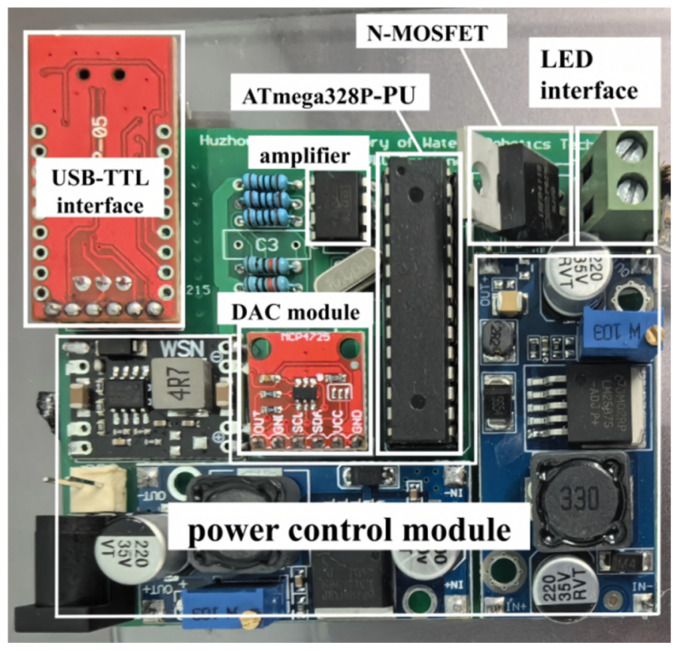
Details of transmitter circuit.

In the Emulation Platform, the optical signal is generated by the transmitter. The central component of this transmitter is thus the LED, which, as detailed in Table 1, has a central wavelength of 450 nm and a semi-angle at half power of 67.5. A pseudo-random binary sequence (PRBS) is generated by a center computer and transmitted to a microcontroller, which interfaces with a digital-to-analog converter (DAC) module via an I2C interface. The DAC provides an analog signal with adjustable amplitude, which is subsequently amplified by an operational amplifier and applied to the gate of an N-MOSFET. The N-MOSFET functions as a switch to regulate the current output of the matrix LED light source. The source and drain terminals of the N-MOSFET are connected to a DC power supply, which can be adjusted according to the light source requirements, allowing for modulation of the DC bias voltage. The physical layout of the transmitter circuit is as illustrated in Figure 4.

Figure 4 illustrates the modular design of the optical transmitter. The system integrates an ATmega328P-PU microcontroller (Microchip Technology, Chandler, AZ, USA), DAC module, amplifier, and N-MOSFET driver for analog signal generation and current modulation. A USB-TTL interface enables communication with the host computer, while a dedicated power-control module regulates the voltage supply. The LED interface provides adjustable optical output for emulating different transmission conditions in the UOCC test bench.

The receiver in the experimental setup is a CMOS sensor, selected based on equipment availability. As detailed in Table 1, the sensor uses an electronic rolling shutter and is identified as model CGU2-500-UVC (LTCAM, Shenzhen, China). It features a resolution of 1920 × 1080 pixels and operates at 30 frames per second. A complete summary of the experimental parameters is provided in Table 1.

The tank allows controlled variation of turbidity (100–400 NTU), flow speed (0.15–1.05 m/s), and ambient illumination, but it cannot capture all features of natural waters. In particular, the particle-size spectrum in the field produces stochastic multi-path scattering that is difficult to reproduce; saline environments introduce wavelength-dependent absorption; and large-scale turbulence exceeds the dimensions and energy of a laboratory tank.

We address these gaps in two ways. First, we apply Real-to-Sim calibration using field measurements to align the simulated channel with observed statistics. Second, we assess external validity through shallow-water tests (Section 4.5). Remaining limitations and their implications for deployment are discussed in Section 5.1.

### 3.2. Datasets Preparation

We generated a diverse dataset in a controlled tank to improve the generalizability of HCMM. Turbidity and flow speed were varied within bounds informed by field observations: nearshore waters often show fluctuations up to ±20% in turbidity and ±5–10% in flow due to wind, waves, and vessel traffic [41,42,43]. To balance realism and repeatability, we adopted a conservative ±10% variation when sampling conditions and used these settings to test model stability.

The dataset construction assumed (i) a quasi-uniform particle distribution during turbidity control, (ii) negligible polarization and wall reflections due to tank geometry and black lining, (iii) constant water temperature over each session, and (iv) fixed camera intrinsics across sessions. These assumptions may shift absolute SNR values but should not affect the relative ranking of methods. This protocol provides realistic yet reproducible conditions for evaluating model robustness.

**Figure 5:**
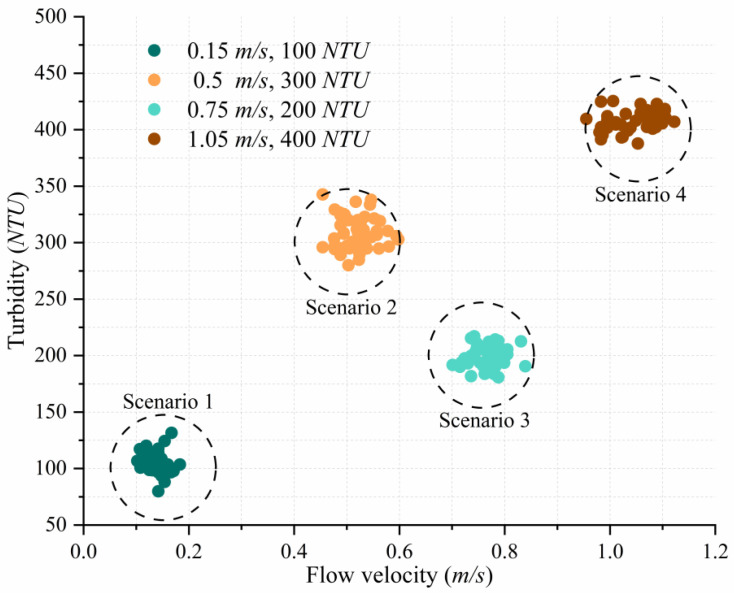
The changes in turbidity and flow velocity in four underwater scenarios.

Four representative underwater conditions were designed as benchmark scenarios: (1) 0.15 m/s, 100 NTU; (2) 0.75 m/s, 200 NTU; (3) 0.5 m/s, 300 NTU; and (4) 1.05 m/s, 400 NTU. For each condition, turbidity and flow velocity were continuously recorded over a 20-s period using high-precision sensors. As illustrated in Figure 5, temporal fluctuations in both parameters remained within the predefined ±10% margin, confirming that the simulated disturbances align with the stochastic behavior of real aquatic environments. Minor variations in turbidity were primarily caused by sediment redistribution, while flow variability reflected transient hydrodynamic effects. It is important to note that HCMM performance under extreme or untested conditions—such as turbidity beyond 400 NTU or high-turbulence scenarios—has not yet been evaluated. Future work should extend the dataset to include such challenging cases, improving the HCMM’s robustness and generalization in more severe underwater environments.

Figure 5 details the four benchmark underwater scenarios used for training/validation, shown as compact clusters in the flow-velocity–turbidity plane. Dashed circles indicate ±10% variability, confirming stable and repeatable emulation. For each scenario, four optical signal power levels were tested: 0.1 W, 0.2 W, 0.3 W, and 0.4 W. Experiments were conducted under two ambient lighting conditions: standard indoor illumination (96.57 lumens, measured by the ambient light sensor) and complete darkness (0 lumens). This resulted in a total of 32 distinct experimental conditions (four scenarios × four power levels × two lighting settings).

The CMOS camera used in the experiments supports 14 discrete exposure values (−13 to 0) and 16 ISO settings (1 to 16). For each environmental condition, all 224 combinations were captured, resulting in 224 images per setup. Across 32 environmental configurations, a total of 7168 RGB images were collected. For each image, metadata—including exposure time, ISO setting, and environmental parameters—were recorded in a structured CSV file. Images were saved in PNG format and organized into clearly labeled directories for downstream analysis. The dataset was split into training, validation, and test sets in a 70%:15%:15% ratio. A batch size of 32 was used, and training was conducted for up to 100 epochs. To enhance training stability and generalization, dynamic strategies were employed, including validation-based monitoring, adaptive learning rate scheduling, and early stopping. Performance metrics were logged at each epoch, and the HCMM with the lowest validation loss was saved in “.pth” format as the final output.

### 3.3. Implementation Details

To train the HCMM for the dual-output task of predicting optimal exposure time and ISO, the Mean Squared Error (MSE) loss function is employed. MSE quantifies the average squared difference between the predicted and ground truth values, offering a direct and interpretable objective for optimizing regression performance. It is formally defined as

(11) $$L_{MSE}=1/N\sum_{j=1}^{N}\left({\left({\hat{e}}_{j}-e_{j}^{*}\right)}^{2}+{\left({\hat{i}}_{j}-i_{j}^{*}\right)}^{2}\right)$$

where ${\hat{e}}_{j}$, ${\hat{i}}_{j}$ denote the HCMM’s predicted values, and $e_{j}^{*}$, $i_{j}^{*}$ represent the experimentally determined optimal exposure time and ISO sensitivity for the *j*-th training sample. Models were implemented in PyTorch (Version: 2.7.1) with CUDA 12. * and trained on an NVIDIA RTX 4060 Ti (16 GB, ASUS, Santa Clara, CA, USA) in a workstation with an Intel i9-13900K and 32 GB RAM. Images (1920 × 1080) were resized to 224 × 224 to match ResNet50 and normalized with ImageNet statistics to align with the pretrained backbone. CPU-only deployment profiling is reported in Section 4.6.

To ensure stable convergence and mitigate overfitting, the Adam optimizer was used with a hybrid learning rate scheduling strategy. The learning rate was initially set to 1 × 10^−4^ and held constant for the first 30 epochs to support broad parameter exploration. Afterward, it was reduced to 1 × 10^−5^ to allow finer updates. A dynamic adjustment mechanism, ReduceLROnPlateau, monitored validation loss and halved the learning rate if no improvement was observed over three consecutive epochs, with a lower bound of 1 × 10^−7^. Training was terminated early if three such reductions failed to yield further improvement, enabling the HCMM to avoid suboptimal minima and supporting convergence in non-convex optimization landscapes.

To further prevent overfitting, early stopping was employed with a patience threshold of 10 epochs. Training halted if validation loss failed to improve over 10 consecutive epochs, and the HCMM with the lowest validation loss was retained. Dropout regularization with a rate of 0.2 was applied within the fusion MLP to enhance generalization.

### 3.4. Evaluation Metrics

To evaluate the HCMM’s ability to predict optimal exposure and ISO settings under complex underwater conditions, a set of quantitative metrics was used to assess both overall accuracy and error distribution. Specifically, Mean Absolute Error (MAE) and Root Mean Square Error (RMSE) were adopted as standard regression metrics. These measures provide a clear assessment of prediction performance and are defined as follows:

(12) $$MAE=1/N\sum_{j=1}^{N}\left(({\hat{e}}_{j}-e_{j}^{*})+({\hat{i}}_{j}-i_{j}^{*})\right)$$

(13) $$RMSE=\sqrt{1/N\sum_{j=1}^{N}\left({\left({\hat{e}}_{j}-e_{j}^{*}\right)}^{2}+{\left({\hat{i}}_{j}-i_{j}^{*}\right)}^{2}\right)}$$

To analyze the statistical distribution of prediction errors across the validation set, the Cumulative Distribution Function (CDF) of absolute errors was plotted. For each sample, the mean absolute error was calculated by averaging the prediction errors of both exposure and ISO:

(14) $$\varepsilon_{i}=1/2(\left|{\hat{e}}_{j}-e_{j}^{*}\right|+\left|{\hat{i}}_{j}-i_{j}^{*}\right|)$$

The scalar errors ${\left\{\varepsilon_{i}\right\}}_{i=1}^{N}$ were sorted in ascending order and plotted against their cumulative probabilities to generate the CDF curve. This visualization reflects the HCMM’s robustness—steeper curves indicate that predictions are closely clustered around the ground truth. From the CDF, key statistics such as the 90th percentile error were extracted, representing the maximum error within which 90% of the predictions fall, thereby providing a probabilistic bound on worst-case performance.

## 4. Results and Discussion

### 4.1. Training Performance

**Figure 6:**
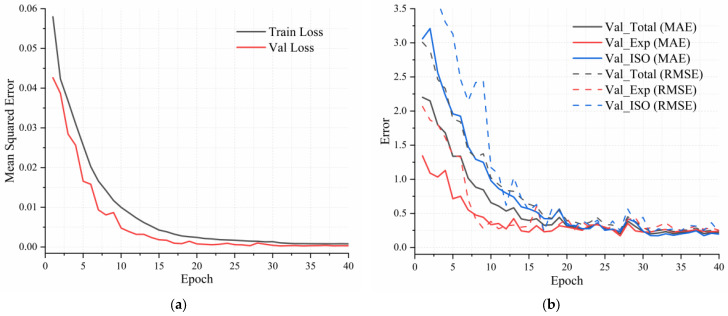
Training performance of the proposed HCMM model: (**a**) Training and validation loss curves across 40 epochs; (**b**) Denormalized error analysis on the validation set, including MAE and RMSE for exposure, ISO, and total prediction error.

To evaluate the overall effectiveness of the proposed multimodal framework, we first analyze training dynamics and predictive performance on the validation set. As shown in Figure 6a, both training and validation losses decrease steadily over 40 epochs, converging to a mean squared error (MSE) below 0.002. This trend indicates a stable optimization process with effective regularization and minimal overfitting.

Additional insights are provided in Figure 6b, which presents the denormalized Mean Absolute Error (MAE) and Root Mean Square Error (RMSE) for both exposure time and ISO predictions, along with the total error. While early training stages show higher variance due to environmental diversity, the HCMM stabilizes quickly—achieving a total MAE below 0.25 by epoch 30. Notably, exposure predictions consistently outperform ISO, suggesting that visual features offer stronger cues for exposure estimation, whereas ISO is more sensitive to environmental conditions.

Table 2 summarizes the quantitative results from the final training phase. The HCMM achieves its best validation performance at epoch 34, with a total MAE of 0.202—comprising 0.225 for exposure and 0.179 for ISO—and a corresponding total RMSE of 0.2349. These results demonstrate the HCMM’s high precision in estimating optimal imaging parameters under varying underwater conditions.

### 4.2. Ablation Study

To evaluate the contribution of each input modality—image features, environmental data, and camera parameters—we performed ablation studies by selectively disabling branches of the proposed architecture. Three model variants were tested:

1. HCMM (FullModel): the complete framework with all three inputs (image, environment, and camera state);
2. NoCam: excludes camera parameters (current exposure and ISO);
3. NoEnv: excludes environmental inputs (turbidity, flow, ambient light, and transmission power).

**Figure 7:**
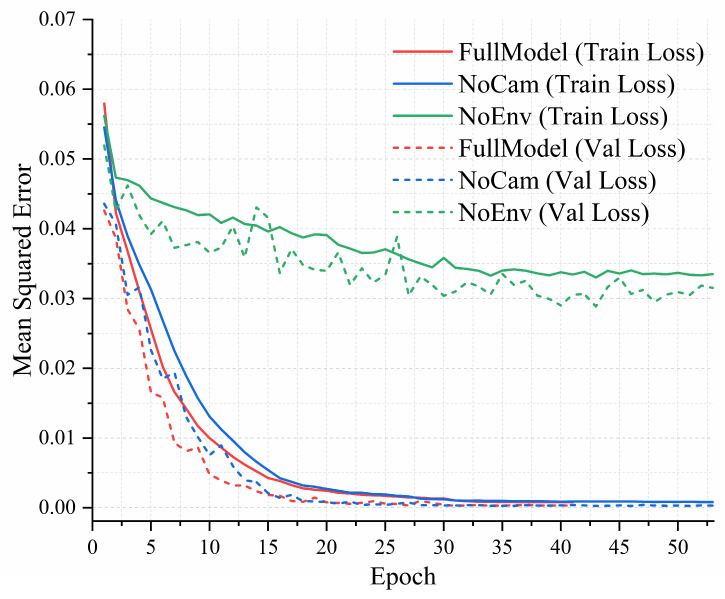
Training and validation mean squared error (MSE) curves for the three model variants in the ablation study: FullModel (HCMM, red), NoCam (blue), and NoEnv (green). Solid lines represent training loss, and dashed lines represent validation loss.

Training and validation MSE curves for each model are shown in Figure 7, with corresponding quantitative results at epochs 10, 20, 30, 40, and 50 summarized in Table 2. The HCMM exhibits the most stable and accurate performance, reaching a validation loss below 0.0032 within 30 epochs and converging to approximately 3.2 × 10^−4^, demonstrating its effectiveness in jointly predicting exposure and ISO values.

As shown in Table 3, the NoEnv model exhibits substantially higher training and validation losses across all epochs, plateauing around 0.03–0.04. This highlights the importance of environmental inputs in capturing light disturbances and turbidity-driven signal degradation, which are critical for accurate ISO estimation. The NoCam variant converges more quickly than NoEnv and achieves moderate accuracy but consistently underperforms compared to the HCMM, particularly in early epochs. This suggests that camera parameters provide valuable priors that help stabilize the optimization process.

The NoEnv model shows not only slower convergence but also high variance in the loss curves, indicating limited generalization capability. In contrast, removing the camera parameter branch (NoCam) results in a modest increase in final error but does not substantially affect convergence, suggesting that the model can partially compensate using visual and environmental inputs. Overall, the ablation results confirm that the three input modalities are complementary and jointly essential for accurate exposure and ISO prediction.

### 4.3. Inference

To assess real-world performance, the HCMM’s absolute prediction error was evaluated on the held-out test set. As shown in Table 4, the HCMM achieves a total Mean Absolute Error (MAE) of 0.396 and a Root Mean Square Error (RMSE) of 0.326, indicating low deviation from ground truth imaging parameters. Specifically, the exposure prediction attains an MAE of 0.220 and an RMSE of 0.248, while the ISO prediction achieves an MAE of 0.177 and an RMSE of 0.211. These results demonstrate that both exposure and sensor gain can be accurately inferred from multimodal inputs under realistic environmental variations.

**Figure 8:**
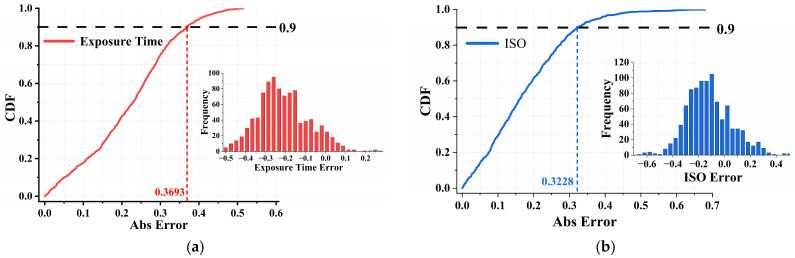
Cumulative distribution functions (CDFs) of absolute prediction errors on the test set: (**a**) Exposure time prediction error distribution, with over 90% of predictions within ±0.37 EV; inset shows histogram of exposure errors; (**b**) ISO prediction error distribution, with over 90% of predictions within ±0.32 levels; inset shows histogram of ISO errors.

The cumulative distribution functions (CDFs) of absolute error are shown in Figure 8a for exposure and Figure 8b for ISO. In both cases, over 90% of predictions fall within ±0.37 EV for exposure and ±0.32 ISO levels. This high prediction accuracy demonstrates the HCMM’s suitability for real-time deployment on AUV systems, enabling dynamic camera adjustment under varying underwater conditions. The narrow error margins and low RMSE values further indicate strong generalization beyond the training domain, validating the effectiveness of the Real-to-Sim transfer strategy.

### 4.4. Baseline Comparison

To comprehensively evaluate the effectiveness of the proposed multimodal prediction framework, we compare it against two representative baselines:

1. Fixed Parameters—a static exposure and ISO configuration applied uniformly across all conditions.
2. CNN-Only Baseline—our previously published CNN-based image–parameter fusion framework [26], which employs ResNet50 with a parameter-aware regression head. This baseline demonstrated the feasibility of learning-based parameter tuning, achieving an RMSE of 0.96 and improving synthetic SNR from 4.16 dB to 6.91 dB.

**Figure 9:**
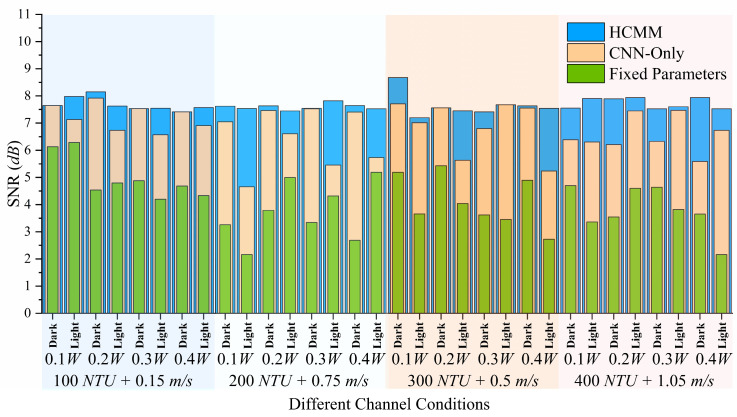
Comparison of the proposed multimodal prediction framework (HCMM) with two baselines under 32 underwater channel conditions. While the CNN-Only Baseline exhibits noticeable instability in certain environments, the HCMM compensates for these fluctuations and consistently stabilizes SNR, highlighting the advantage of environment-aware multimodal learning for practical UOCC deployment.

The proposed HCMM was evaluated under all 32 channel conditions defined in Section 3.2. As illustrated in Figure 9, the Fixed Parameters method results in the lowest and most variable SNR values, reflecting its inability to adapt to environmental changes.

We evaluate all methods under 32-channel settings spanning turbidity, flow, illumination, and source power. Fixed parameters yield the lowest and most variable SNR, confirming that static settings cannot accommodate changing water conditions. The CNN-Only model improves mean SNR but shows pronounced drops in high-turbidity or low-light scenes, indicating limited generalization beyond its training distribution.

HCMM maintains higher and more stable SNR across all 32 conditions. By integrating three signals—image content, environmental measurements, and current camera state—HCMM anticipates scene difficulty and selects safer operating points. As shown in Table 5, this multimodal design reduces parameter-prediction error (test RMSE 0.23–0.33) and raises average SNR to 7.64 dB, a ~1.7 dB gain over CNN-Only. The margin in our tank experiments is smaller than in synthetic studies because turbulence and lighting fluctuations are less severe in the lab, which compresses performance gaps. Even so, HCMM consistently shows the tightest SNR spread, highlighting improved stability.

We also position HCMM against broader multimodal and reinforcement-learning strategies. Recent multimodal OCC systems often merge image and signal features but omit explicit environment embeddings, which hampers robustness under domain shifts. RL controllers can adapt online but typically require many rollouts and a reward signal, increasing latency and energy on embedded platforms. In contrast, HCMM makes one-shot, feed-forward predictions using visual, environmental, and camera-state cues. This keeps inference fast (see Section 4.6) while improving resilience to unseen turbidity and illumination—properties that are particularly important for AUV deployment.

### 4.5. Underwater Measurement and Visualization

While the laboratory experiments verified the relative advantages of HCMM, the controlled environment inherently reduced turbulence and illumination variability, thereby compressing the performance gap between models. To further validate the generalization ability of the proposed framework under more realistic conditions, we conducted additional tests in an untrained shallow-water environment.

**Figure 10:**
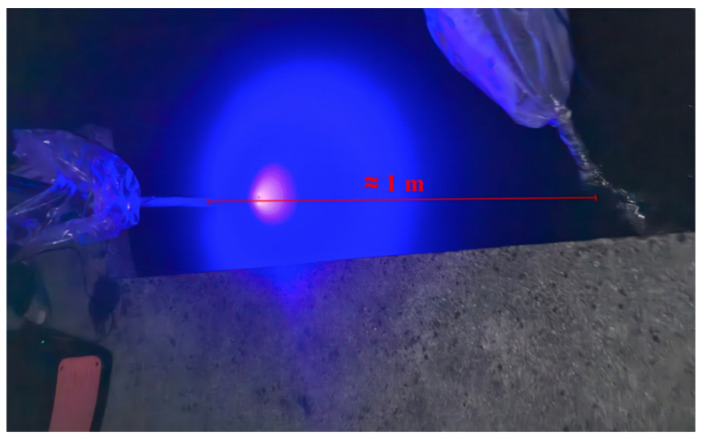
Underwater measurement setup and deployment site at Deqing Lake. The simplified PMMA housing enabled shallow-water testing to verify the generalization ability of HCMM in real-world conditions.

Due to experimental constraints, a simplified waterproof enclosure was developed for the UOCC transceiver system described in Section 3.1. A custom underwater housing was constructed using a transparent acrylic (PMMA) bar-shaped container with an open-top design, chosen to minimize optical distortion while ensuring water resistance. The LED emitter and CMOS receiver were mounted on a rigid vertical rod and inserted into the container, which measured approximately 1 m in length. All sidewalls were made of optically clear PMMA to ensure high light transmittance and low scattering. As illustrated in Figure 10, the open-top design facilitated flexible placement and retrieval while preserving a stable optical interface through the front-facing walls. During deployment, the entire unit was submerged to a depth of approximately 0.5 m, with the upper portion of the container remaining above water to protect the internal electronics. This setup enabled stable testing conditions in shallow water while maintaining consistent sensor alignment.

**Figure 11:**
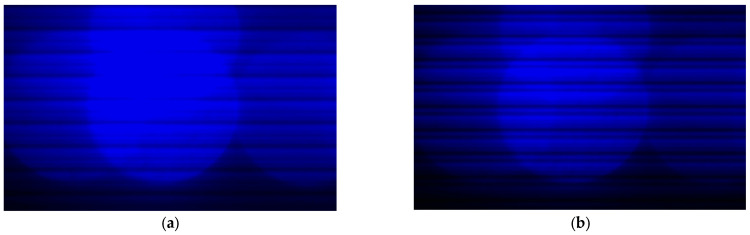
CMOS received image: (**a**) Before; (**b**) After HCMM-guided adjustment. The optimized image exhibits enhanced contrast and reduced noise, highlighting HCMM’s ability to improve visual quality in real underwater conditions.

Field testing was conducted at night along the Deqing Lake shore of Huzhou University. During the experiment, two operators submerged the transceiver setup to a depth of approximately 0.5 m by inserting the mounting pole about 1 m into the water, ensuring proper alignment between the LED transmitter and CMOS receiver. Environmental measurements—including turbidity, flow velocity, and ambient light—were recorded at the same depth using the sensors described in Section 3.1. The corresponding environmental variables and transmitter signal power used as model inputs are summarized in Table 6. Captured images were processed in real time, and the camera parameters were adjusted based on predictions from the trained HCMM. The original image and the optimized image are shown in Figure 11.

**Figure 12:**
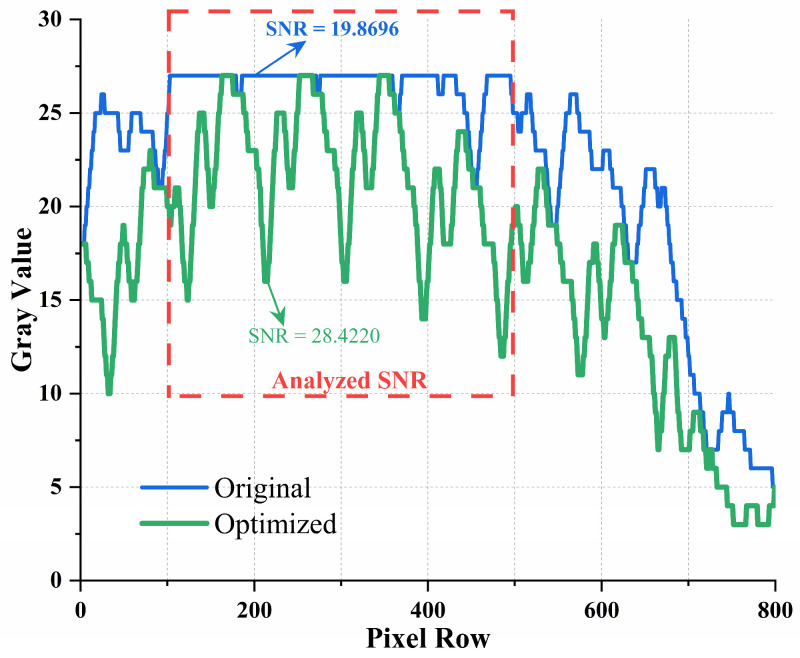
Gray-value analysis of the LED projection region. Compared with the original image (blue), the HCMM-optimized result (green) exhibits significantly higher and more stable SNR, demonstrating improved robustness in real-world underwater conditions.

In UOCC systems, the LED-emitting region captured by the image sensor is the primary area of interest, as it contains the modulated optical signal in the form of spatial fringe patterns. Therefore, image quality metrics such as signal-to-noise ratio (SNR) are most appropriately evaluated within this illumination zone. In our experiments, we focused on the central column pixel range (150–500), which corresponds to the LED’s optical projection region. Gray values from this region were extracted for both the original and HCMM-optimized images, as shown in Figure 12.

The SNR of the original image was approximately 19.87, while the optimized image achieved an SNR of 28.42—indicating a substantial improvement. These results confirm that HCMM not only improves average SNR but also mitigates the instability exhibited by baseline methods in certain conditions, thereby enhancing robustness across varying underwater environments.

Overall, the field tests confirm the effectiveness of the proposed HCMM beyond controlled laboratory conditions. While the performance gain over the CNN-Only Baseline appeared modest in Section 4.4 due to limited turbulence and illumination variability in the emulation platform, the shallow-water deployment demonstrated a much larger improvement in both image quality and SNR (from 19.87 dB to 28.42 dB). These results show that HCMM not only enhances average performance but also compensates for the instability of baseline methods in more realistic environments, reinforcing the importance of environment-aware multimodal learning for practical UOCC deployment.

### 4.6. Computational Efficiency, Deployment, and Concise Synthesis

On a CPU-only host (batch = 1; 224 × 224), HCMM processes a frame in 78.3 ms with 24.76 M parameters (1.25 M trainable) and ~4.13 G multiply–accumulate operations, as shown in Table 7. The CNN-only baseline (same frozen ResNet50 + MLP head) runs in 74.8 ms with 24.72 M parameters (1.21 M trainable) and the same arithmetic cost. The ~3.5 ms difference stems from lightweight branch concatenation and the fusion MLP, not from the backbone.

Ablation (Section 4.5) indicates that visual features dominate exposure prediction, while environment inputs (turbidity and illumination) contribute more to ISO selection—consistent with imaging physics and enabling branch-level constraints in safety-critical modes. Because the backbone is frozen and the fusion head is small, the model exports cleanly to ONNX/TensorRT and permits branch-level pruning (e.g., dropping the camera-state branch) with <2% accuracy loss on our data.

Implication. With CNN-equivalent arithmetic complexity, HCMM delivers more stable link performance via environment awareness and real-data calibration; compared with RL controllers that require online interaction, single-shot inference offers clear latency and energy advantages for constrained deployments.

## 5. Discussion

### 5.1. Expanded Interpretation and Implications

The Real-to-Sim-to-Deployment pipeline helps explain HCMM’s ability to generalize. By calibrating the emulator to field distributions of turbidity, flow, and illumination, the training data span the operating range typical of shallow water. Within this setting, the model assigns complementary roles to its inputs: visual features dominate exposure prediction—consistent with exposure’s dependence on scene contrast—while environment features contribute more to ISO selection, reflecting ISO’s role in compensating for limited photons and variable background light.

From a system viewpoint, stable SNR across changing conditions is more valuable than occasional peaks, especially for swarm AUVs whose decoders can tolerate slight sub-optimality but are vulnerable to sharp SNR dips. HCMM’s lower variance, therefore, translates into fewer packet losses and less frequent link renegotiation. The larger gains observed in shallow-water tests, compared with the lab, indicate that environmental awareness is particularly effective under non-stationary disturbances such as surface waves, suspended sediments, and specular glints. Practically, this supports robust short-range links for cooperative inspection and positioning, where visual sensors are already available and power margins are tight.

### 5.2. Limitations and Future Work

The tank cannot reproduce full multi-path scattering or wavelength-selective absorption; training used a single 470 nm source, which may limit spectral transfer; validation covered 100–400 NTU and moderate flows; and inference profiling focused on CPUs and Jetson-class GPUs rather than ultra-low-power MCUs. These factors may compress or magnify SNR gains relative to harsher open-water settings.

Future work will extend to blue–green bands, integrate Monte-Carlo scattering into Real-to-Sim calibration, and study duty-cycling and quantization for energy-aware AUV autonomy. We will also explore multi-frame temporal fusion, proactive control via reinforcement learning, and end-to-end training that ties exposure decisions directly to communication performance.

## 6. Conclusions

This work presented a Real-to-Sim-to-Deployment framework that integrates a physically calibrated underwater emulation platform with a multimodal deep learning model for adaptive imaging parameter control in underwater optical camera communication (UOCC). The sensor-integrated testbed, built with 470 nm blue LEDs and CMOS cameras under tunable turbidity and flow conditions, enabled reproducible optical channel states and systematic dataset generation. On this basis, we developed a Hybrid CNN–MLP Model (HCMM) that jointly learns from optical images, environmental sensor inputs, and camera configurations. Compared with our prior CNN-based image–parameter fusion baseline—which achieved an RMSE of 0.96 and improved synthetic SNR from 4.16 dB to 6.91 dB but lacked explicit environmental awareness—the HCMM substantially enhances prediction accuracy and robustness. Specifically, it reduces RMSE to 0.23–0.33 and achieves up to 8.5 dB SNR gain in both controlled and field environments, consistently surpassing static-parameter systems and the baseline CNN model. These findings validate the effectiveness of environment-aware multimodal learning in bridging the gap between proof-of-concept feasibility and practical deployment. The proposed framework demonstrates scalable potential for real-time, energy-efficient UOCC on resource-constrained AUV platforms and can be extended to related domains such as underwater positioning, inspection, and laser-based links.

## Figures and Tables

**Table 1:** Parameter configuration.

**Transmitter Characteristics**	
**Parameter**	**Value**
Light Wavelength	450 nm
Photoelectric Conversion Efficiency	65.16%
Half-power Angle	67.5°
**Receiver characteristics**	
**Parameter**	**Value**
Effective pixel	1920 × 1080
Sensor Size	1/2.5″ CMOS
Pixel Size	2.2 UM × 2.2 UM
FPS	30
Contrast	0
Brightness	0
Dynamic Range	70.1 dB
**Channel characteristics**	
**Parameter**	**Value**
Emission Angle	0°
Distance	2 m
Receiving Angle	0°

**Table 2:** Statistical analysis of HCMM prediction errors under different epochs.

	MSE		MAE			RMSE		
Epoch	TrainLoss	ValLoss	Val_Total	Val_Exp	Val_ISO	Val_Tota	Val_Exp	Val_ISO
30	0.00135	4.40655 × 10^−4^	0.23353	0.22686	0.24019	0.29402	0.27461	0.44152
34	8.63233 × 10^−4^	2.92663 × 10^−4^	0.202	0.22541	0.17859	0.23495	0.28077	0.27133
40	8.16385 × 10^−4^	3.18654 × 10^−4^	0.21663	0.23765	0.19560	0.24593	0.25167	0.22916

**Table 3:** The performance of each variant on the validation set at different epochs.

Epoch	HCMM	NoCam	NoEnv
10	0.00478	0.00755	0.03659
20	7.84 × 10^−4^	8.41 × 10^−4^	0.03399
30	4.41 × 10^−4^	3.32 × 10^−4^	0.03041
40	3.19 × 10^−4^	3.42 × 10^−4^	0.02901
50	/	3.29 × 10^−4^	0.03095

**Table 4:** Experimental results on the test set.

Indicator Type	Exposure Time (EV Level)	ISO Value (Level)	Overall (Exp + ISO)
MAE	0.2199	0.1765	0.3964
RMSE	0.2479	0.2110	0.3256

**Table 5:** Performance comparison of different baseline and proposed models.

Method	RMSE (Parameter Prediction)	Average SNR (dB)	Remarks
Fixed Parameters	– (no learning)	~4.0 (highly variable)	Uses static exposure & ISO across all conditions; lowest robustness.
CNN-Only Baseline	0.96 (reported)	6.91 (synthetic)	Image + parameter fusion; validated feasibility but lacks environmental awareness.
HCMM (Proposed)	0.23–0.33 (test set, Table 4)	7.64 (stable)	Multimodal (image + environment + camera state); highest accuracy and stability.

**Table 6:** Underwater measurement environment variables.

Parameter	Value
Turbidity (NTU)	≈38
Flow velocity (m/s)	≈0.08
Transmitter signal power (W)	0.4
Ambient light (lm)	≈0

**Table 7:** Concise synthesis of outcomes and relation to prior work (CPU-only; parameters in millions; arithmetic cost from THOP).

Outcome	Our Result	Baseline/Prior	Alignment or Anomaly
EV/ISO prediction	Low RMSE; >90% within tight bounds	Higher RMSE (CNN-only)	Consistent: multimodal > image-only
SNR (lab, 32 conditions)	Higher mean, lower variance	Fixed/CNN-only less stable	Consistent: adaptive > static; stability matters
SNR (shallow water)	Larger gains than in tank	—	Explained: stronger field disturbances → larger benefit
Inference cost	78.3 ms vs. 74.8 ms; both ~4.13 G MACs	Same GMACs	Consistent: fusion overhead negligible
Control strategy	Feed-forward; no online rollouts	RL requires online rollouts [19]	Different: latency/energy advantage

## Data Availability

The data that support the findings of this study are available from the corresponding author upon reasonable request.
